# 3,3’-((3,4,5-trifluoropHenyl)methylene)bis(4-hydroxy-2H-chromen-2-one) inhibit lung cancer cell proliferation and migration

**DOI:** 10.1371/journal.pone.0303186

**Published:** 2024-05-22

**Authors:** Wenhui Luo, Guoxin Chang, Dingmei Lin, Hongyi Xie, Huilong Sun, Zhibin Li, Shirong Mo, Ruixue Wang, Yan Wang, Zhaoguang Zheng

**Affiliations:** 1 School of Medicine, Foshan University, Foshan, Guangdong Province, PR China; 2 Guangdong Provincial Key Laboratory of Traditional Chinese Medicine Formula Granule, Guangdong Yifang Pharmaceutical Co., Ltd., Foshan, Guangdong Province, PR China; 3 College of Traditional Chinese Medicine, Guangdong Pharmacuetical University, Guangzhou, Guangdong Province, PR China; Kafrelsheikh University Faculty of Pharmacy, EGYPT

## Abstract

Lung cancer is a major public health challenge and, despite therapeutic improvements, is the first leading cause of cancer worldwide. The current cure rate from advanced cancer treatment is excessively low. Therefore, it is of great importance to identify novel, potent and less toxic anticancer agents for the treatment of lung cancer. The aim of our research is to synthesize a new biscoumarin 3,3’-((3,4,5-trifluorop -phenyl)methylene)bis(4-hydroxy-2H-chromen-2-one) (C35) as an anticancer agent. C35 was simply prepared by 4-hydroxycoumarin and 3,4,5-trifluorobenzaldehyde under ethanol and its structure was analyzed by spectroscopic analyses. The anti-proliferation effect of C35 was detected using CCK-8 assay. Migration abilities were measured by Transwell assay. The expression of correlated proteins was determined by Western blot. The results showed that C35 displayed strong cytostatic effects on lung cancer cell proliferation. In addition, C35 possessed a significant inhibition of migration by reducing the expression of matrix metalloproteinases-2 (MMP-2) and MMP-9 in lung cancer cells. Furthermore, C35 treatment suppressed the phosphorylation of p38 in lung cancer cells. Moreover, in vivo experiments were carried out, in which we treated Lewis tumor-bearing C57 mice via intraperitoneal injection of C35. Results showed that C35 inhibited tumor growth in vivo. In conclusion, our study demonstrated the anticancer activity of C35 via suppression of lung cancer cell proliferation and migration, which is possibly involved with the inhibition of the p38 pathway.

## Introduction

Lung cancer is the most common cause of cancer death, accounting for 24% of cancer-related deaths, with a 5-year survival rate of only 15% after diagnosis. Treatment options for lung cancer include surgery, chemotherapy, radiotherapy, immunotherapy and targeted therapy. In recent years, significant progress has been made in immunotherapy and targeted therapy for lung cancer, but the 5-year survival rate of lung cancer patients is still low, and lung cancer is prone to recurrence and metastasis. Therefore, it is of great importance to identify novel, potent, and less toxic anticancer agents for lung cancer treatment.

The natural products are important sources of drugs with a significant proportion of current drugs being natural products or derived from natural products. Coumarin, also known as 1,2-benzopyranone, has a large conjugated system in which the benzene ring and the pyranone ring in its structure can form a large conjugated system, making coumarin highly modifiable and capable of introducing a variety of functional groups [1]. Coumarin-derived compounds obtained through total synthesis or structural modification can enhance their anti-tumor activity. Accumulating data have shown a number of synthesized coumarin-derived compounds for their potential anti-tumor activities [1]. Those coumarins-based anticancer agents have been identified for a variety of mechanisms of action, including alkylating agents, topoisomerase inhibitors, angiogenesis inhibitors, apoptosis inducers, human carbonic anhydrase inhibitors, telomerase inhibitors and miscellaneous agent [1].

Biscoumarin, a coumarin-derived compound, is mainly used as an anticoagulant for the prevention and treatment of thrombosis. Recently the anti-diabetic [2] and anti-tumor [3] effects of biscoumarin have also been reported. Methylenebis (4-hydroxy-2H-chromen-2-one) biscoumarins are easily and simply synthesized by 4-hydroxycoumarin and different substituent benzaldehydes. Different substituent benzaldehydes affect the efficiency of synthesis and the activity of the product. Therefore, the selection of benzaldehyde with different substituents is particularly important. Previously, we found that the synthesized biscoumarin 3,3’-((4-chlorophenyl)methylene)bis(4-hydroxy-2H-chromen-2-one) suppressed non-small cell lung cancer cell proliferation and induced cell apoptosis, possibly involving receptor interacting protein-1 [4]. Fluorine and chlorine are both halogen elements, and the rational design by the introduction of fluorine into a compound has achieved success in the development of organic anticancer drugs [5–7]. So here we synthesized another similar biscoumarin 3,3’-((3,4,5-trifluoropHenyl)methylene)bis(4-hydroxy-2H-chromen-2-one) (compound C35), with three fluorine substituents instead of one chlorine substituent on the benzaldehyde unit, in order to explore more potential candidates for lung cancer treatment.

## Materials and methods

### Chemicals and apparatus

The established H1299 and Lewis cell lines were obtained from The Chinese Center for Type Culture Collection. NMR data were collected on a Bruker AM-400 spectrometer in DMSO-d6 (Bruker, Fällanden, Switzerland). HR-ESI-MS were performed in MeOH on a thermofisher Q-Fleet spectrometer (Thermofisher Scientific, San Jose, CA, USA). The melting point was detected by RY-2 melting point meter (Tianjin analytical instrument factory, Tianjin, China).

### Synthesis of compound biscoumarin (C35)

**Fig 1:**
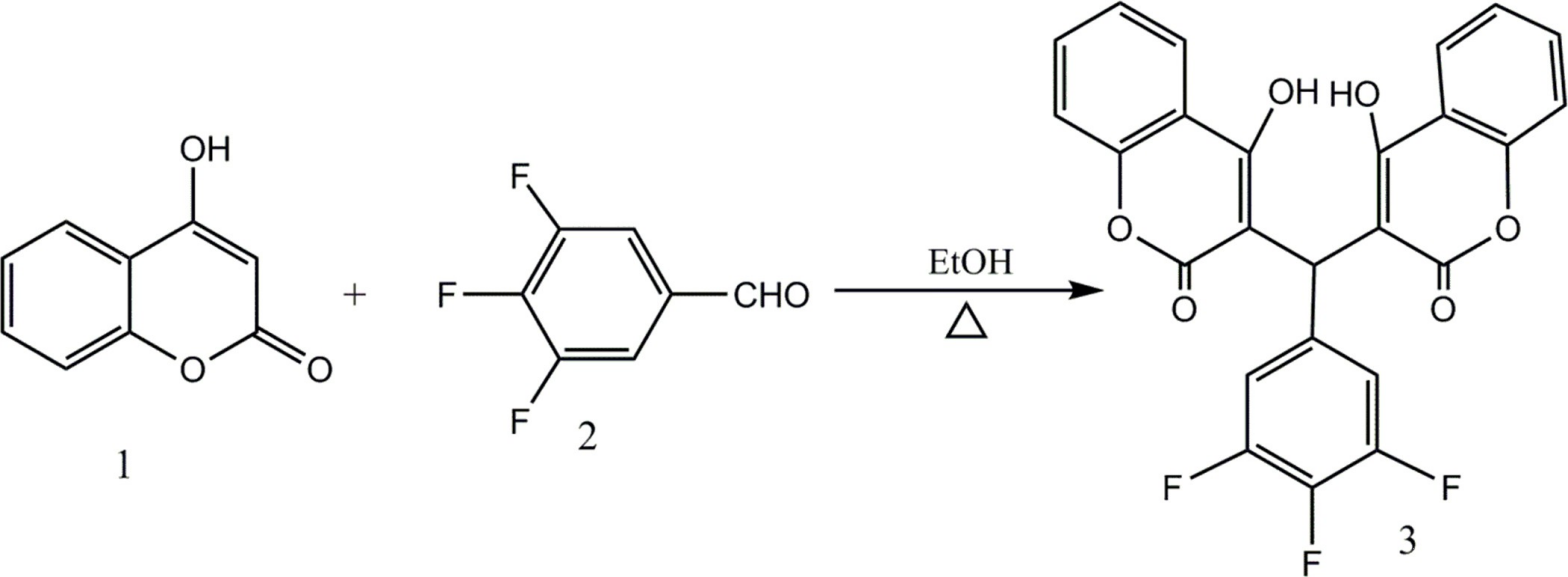
Synthesis of C35.

The synthesis of C35 is according to our previous paper [4]. In detail, 1.62 g (10 mmol) 4-hydroxycoumarin (1) and 5 mmol 3,4,5-trifluorobenzaldehyde (2) were dissolved in 20 mL ethanol, placed in a 50 mL round-bottom flask, stirred and heated at reflux for 4 h, solid precipitation could be seen in the reaction, monitored by thin layer chromatography (TLC) until the end of the reaction, cooled and filtered, the precipitate was recrystallized with ethanol to obtain 3 (C35, Fig 1).

### Cell culture

H1299, Lewis, H9C2 and Beas-2B cells were maintained in Dulbecco’s modified Eagle’s medium (DMEM; Gibco, Waltham, MA, USA) supplemented with 10% fetal bovine serum (FBS; Gibco, Waltham, MA, USA). All cells were cultured at 37°C in a humidified atmosphere containing 5% CO_2_. The cell authentication was performed via STR profiling and species authentication. All the cells were passaged fewer than 20 times.

### Cell proliferation assay

The H1299, Lewis, H2C9 and Beas-2B cells were plated (2 × 10^3^/well) in 96-well plates and incubated with C35 (0, 10, 20 μM) for 72 h, respectively. CCK-8 solutions (Dojindo, Japan) were then added and maintained at 37°C for 1 h. Finally, the absorbance was measured at 450 nm.

### Western blot

Western blot was performed according to our previous paper [4]. In brief, whole cell extracts were prepared by lysing the cells in lysis buffer (KeyGEN biotech, Nanjing, China). Then the equal amounts of total proteins were resolved by SDS-PAGE and the proteins of interest were probed by Western blot. Subsequently the Western blot results were visualized by enhanced chemiluminescence according to manufacturer’s instructions (Millipore, Billerica, MA, USA). The expression of protein was quantified by Image J software.

### Transwell assay

A Transwell chamber (Corning, MA, United States) was used to analyze cell migration capacity. Briefly, the lung cancer cells in serum-free medium were loaded into the upper chamber and the lower chamber was loaded with medium containing 5% FBS. Then the cells were treated with C35 (0, 10, 20 μM). After 24 h of incubation, non-migration cells were removed and migration cells were stained with crystal violet. Finally, the number of migration cells was counted by microscopy.

### ELISA

The amount of MMP-2 and MMP-9 in cell culture supernatants were determined by ELISA according to the manufacturer’s instructions (Zikerbio, Shenzen, China). Briefly, cells were plated on 6-well plates at 70–80% confluence. After overnight culture, cells were treated with C35 (0, 10, 20 μM) for 72 h, then cell culture supernatants were collected and incubated with HRP-labelled detection antibody in 96-well plates. After washing 5 times with washing buffer, 50 μL of substrate A and B were added to each well, followed by 50 μL of stopping solution. OD values were measured at 450 nm.

### In vivo study

Six-week-old male mice were purchased from Animal Center (Guangzhou, China) and maintained under pathogen-free conditions. All procedures involving animals and their care were conducted in in accordance with the guidelines of the Institutional Animal Care and Use Committee of Foshan University. A total of 1× 10^6^ cells in 100uL Phosphate Buffered Saline (PBS) was subcutaneously injected in the right flank of the mice. After palpable tumors had developed, mice were randomly divided into three groups and received four intraperitoneal injections of following agents: (a) PBS control; (b) 50 mg/Kg C35; (c) 75 mg/Kg C35. There are 5 mice in each group. The tumor volumes were measured with a caliper and calculated as the following formula: V = 0.5 × length × width^2^, the length was the long axis of the tumor, and the width was the short axis. At the end of the experiments, mice were euthanized and the excised tumors were collected and weighed.

### Statistical analysis

Data are presented as mean ± SD. Statistical analyses were performed with GraphPad PRISM 6.0 software. Significant differences between two groups were compared using a Student’s t-test (two-tailed). A one-way ANOVA was used in multiple comparisons.

## Results

### Identification of C35

3,3’-((3,4,5-trifluoropHenyl)methylene)bis(4-hydroxy-2H-chromen-2-one) (C-35), 1.89g with the yield of 81%, white power, formula: C_25_H_13_F_3_O_6_, m.p. 250–252°C; ESI-MS, m/z: 467 [M+H]^+^; IR (KBr): 3070, 2725,2585, 1671 (C = O), 1604, 1528, 1434, 1349, 1101 cm^-1^; 1H-NMR (DMSO-d6, 400MHz): d 6.275 (s, 1H, H-11), 7.063 (dd, 2H, J = 6.4, 10.4Hz, H-2′′,6′′), 7.289 (td, 2H, J = 1.2, 8.0 Hz, H-6,6′), 7.338 (dd, 2H, H-8,8′), 7.585 (td, 2H, J = 1.6, 7.6 Hz, H-7,7′), 7.894 (dd, 2H, J = 1.6, 8.0Hz, H-5,5′), 10.685 (brs, 2H, 2OH). Compared to the literature [8], the compound is identified as 3,3’-((3,4,5-trifluoropHenyl)methylene)bis(4-hydroxy-2H-chromen-2-one) (Fig 1, S1-S3 Figs in S1 File).

### C35 inhibited the proliferation of lung cancer cells

**Fig 2:**
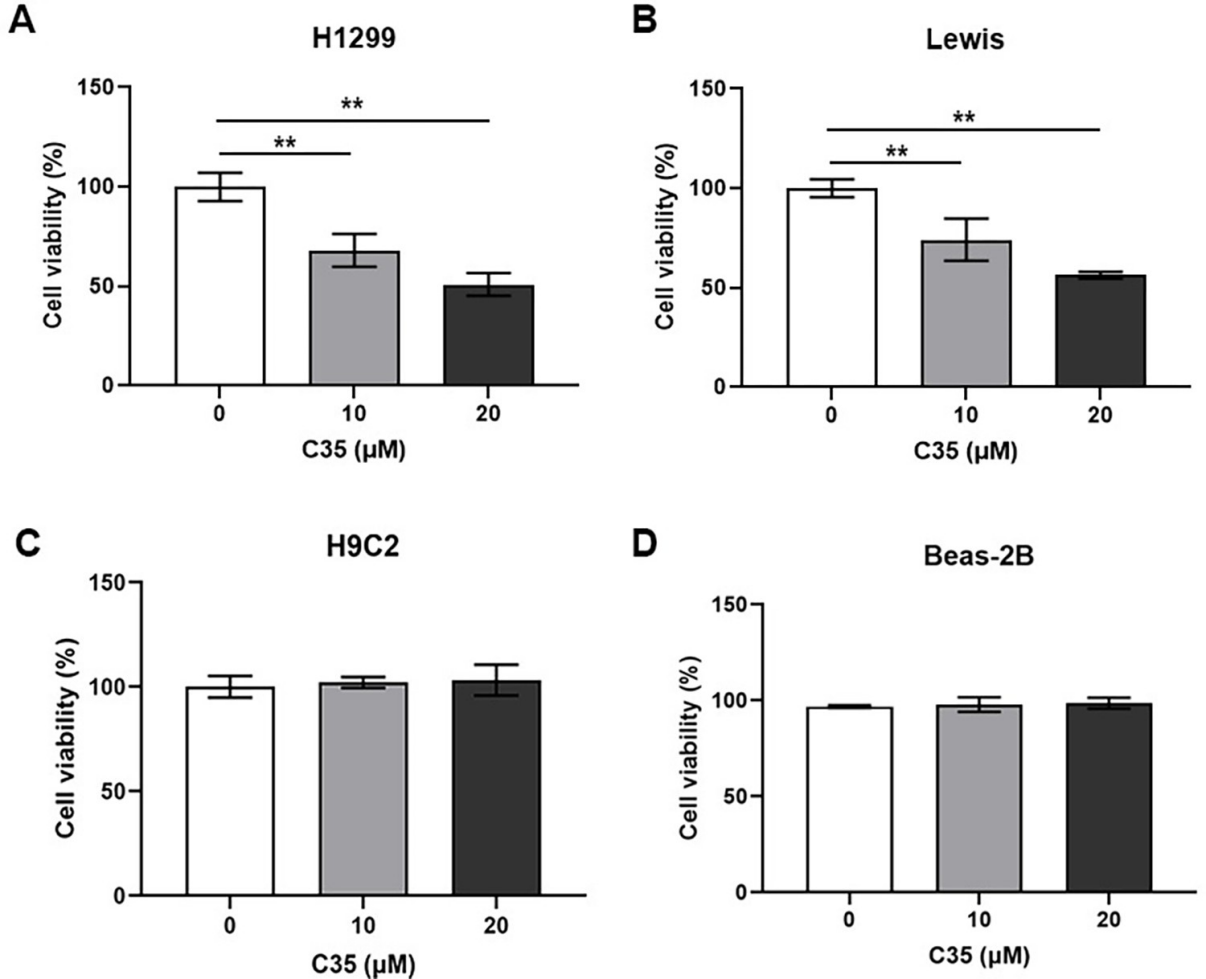
The proliferation of lung cancer cells was inhibited by C35. The H1299 (A), Lewis (B), H9C2 (C) and Beas-2B (D) cells were treated with C35 at the indicated concentrations (0, 10 and 20 μM) for 72 h. And then the cell viability was measured by CCK-8 assay. Data are presented as mean±SD of three independent experiments. **p<0.01.

To evaluate the anti-proliferative effect of C35, human lung cancer cell H1299 and mouse lung cancer cell Lewis cells were treated with various concentrations of C35 for 72 h, respectively, and cell proliferation was measured by CCK-8 assay. We found that C35 inhibited the proliferation of H1299 and Lewis cells in a manner with dose-dependent (Fig 2A and 2B). In H1299 and Lewis, C35 had IC50 values of 20.77μM and 20.87μM, respectively. In addition, the human pulmonary epithelial cell line Beas-2B and normal rat cardiomyocytes cell line H9C2 were used to assess the cytotoxic effect of C35. As shown in Fig 2C and 2D, C35 is less toxic to H9C2 and Beas-2B cells than to H1299 and Lewis cells, which suggests that C35 inhibits lung cancer growth in a specific manner.

### C35 reduced the migration of lung cancer cells

**Fig 3:**
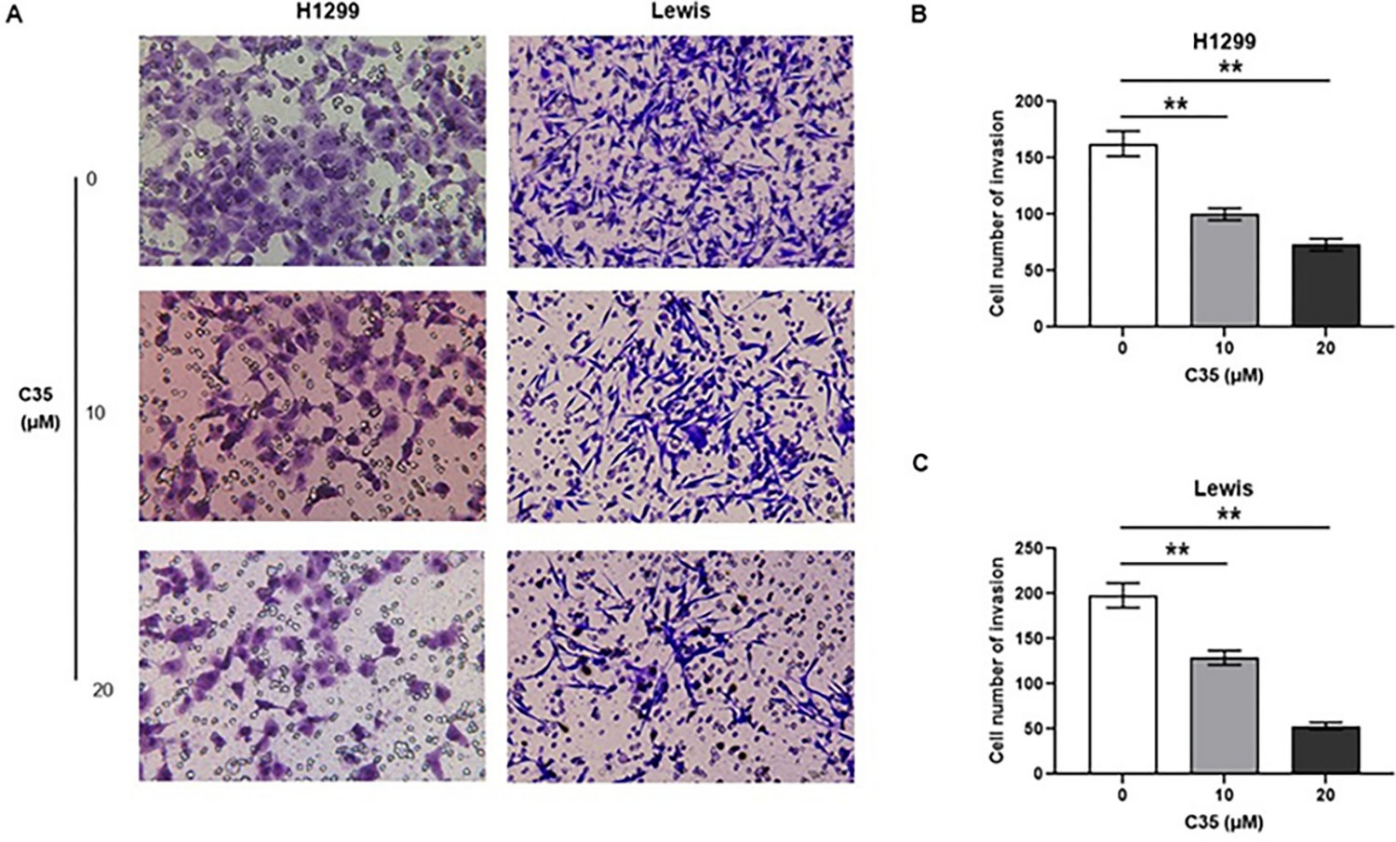
The migration of lung cancer cells was reduced by C35. (A) H1299 and Lewis cells were treated with different concentrations of C35 (0, 10, and 20 μM) for 24 h. (B and C) The migration cells were photographed and quantified. Data are presented as mean±SD of three independent experiments. **p<0.01.

Cell migration is a key step in the cancer progression. To further investigate the role of C35 on cell metastasis, the cells were subjected to an Transwell migration assay. As shown in Fig 3, there is a clear trend towards a dose-dependent decrease in migration cells after C35 treatment in both H1299 and Lewis cells.

**Fig 4:**
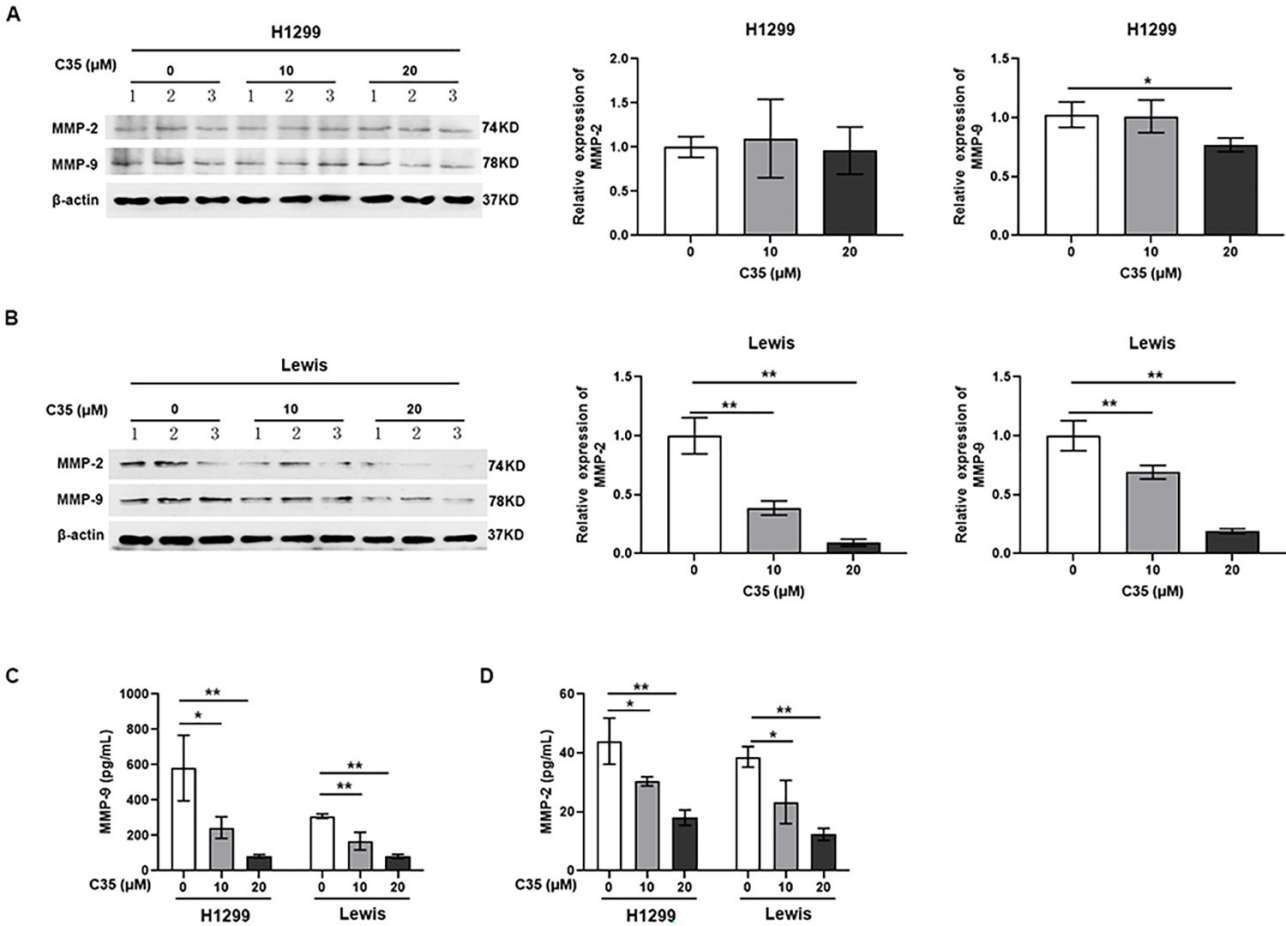
C35 reduced the expression of MMP-2 and MMP-9. (A and B) Western blot analyses of the relative expression of MMP-2 and MMP-9 in H1299 and Lewis cells treated with or without C35 as indicated concentrations. β-actin served as loading control. 1, 2 and 3 represent three of the sample in the indicated group. (C and D) The amount of MMP-2 and MMP-9 in H1299 and Lewis cell culture medias were determined by ELISA. Data are presented as mean±SD of three independent experiments. *p<0.05.**p<0.01.

As tumor cells migrate, MMPs degrade the basement membrane [9]. Therefore, the protein expression of MMP-2 and MMP-9 in lung cancer cells in response to C35 treatment were evaluated through Western blot and ELISA. The Western blot analysis showed that the high concentration of C35 (20 μM) stimulation decreased the expression of MMP-9 in H1299 cells (Fig 4A). In addition, C35 stimulation decreased the expression of MMP-9 and MMP-2 in Lewis cells (Fig 4B). Moreover, our data showed that C35 treatment significantly inhibited the amount of MMP-2 and MMP-9 in extracellular fractions in both H1299 and Lewis cells (Fig 4C and 4D). Taken together, those results suggest that C35 reduces lung cancer cell migration.

### C35 suppressed the phosphorylation of p38 in lung cancer cells

**Fig 5:**
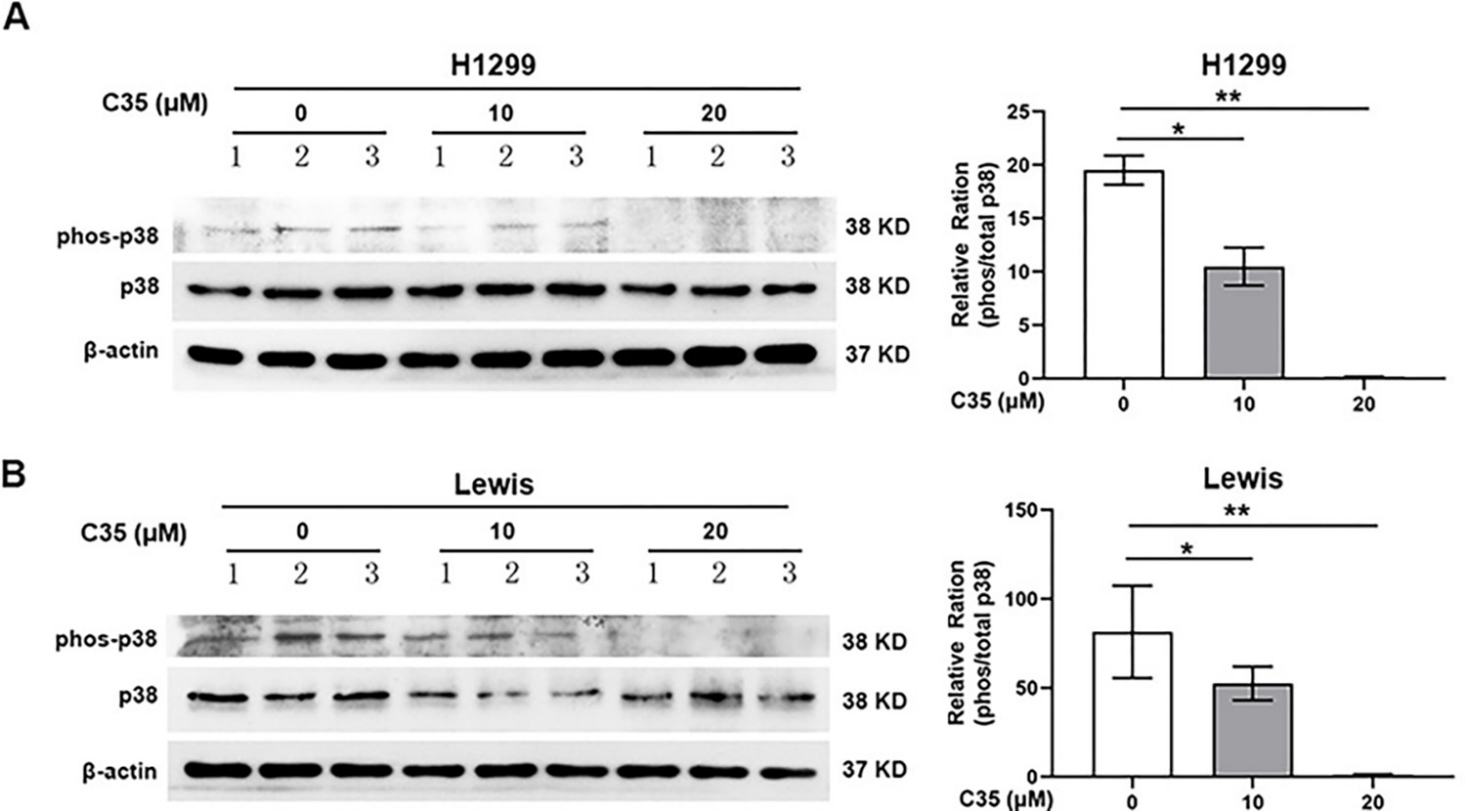
Western blot analyses of the phosphorylation of p38 in H1299 (A) and Lewis (B) cells treated with or without C35 as the indicated concentrations. β-actin served as loading control. The 1, 2 and 3 represent three of the samples in the indicated group. Data are presented as mean±SD of three independent experiments. *p<0.05.**p<0.01.

AKT and p38 pathways has been reported to regulate cell proliferation, migration, and invasion in cancer [10, 11]. Consequently, we investigated whether C35 has an impact on these signaling pathways, and found that it decreased p38 phosphorylation (Fig 5), but not AKT phosphorylation (S4 Fig in S1 File).

### C35 inhibited the tumor growth in vivo

**Fig 6:**
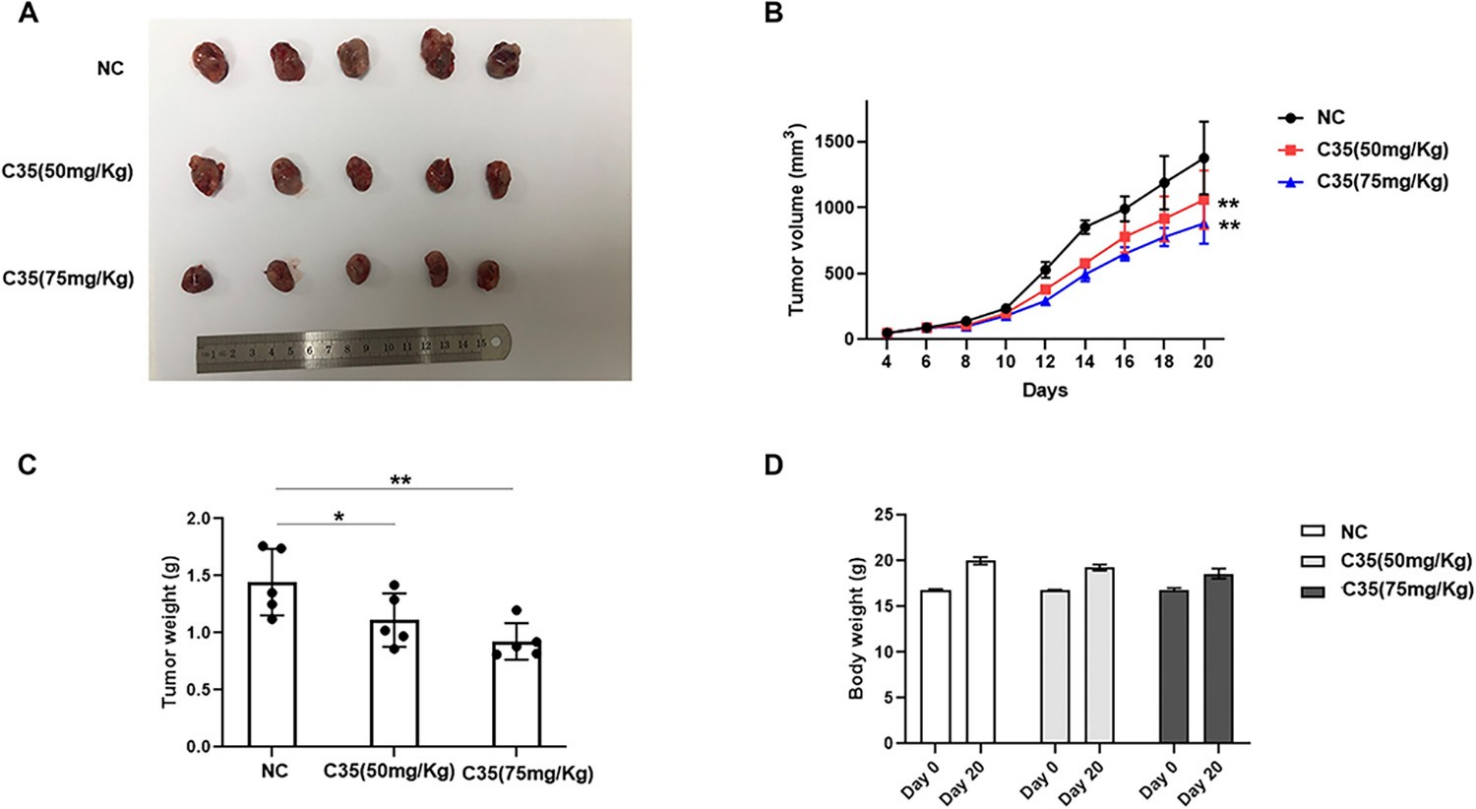
C35 inhibited tumor growth on Lewis allograft tumor in C57 mice. (A) Images of Lewis tumor of C57 mice treated with PBS or C35 (n = 5 in each group). (B) The tumor volume was measured every 2 days, and the tumor growth curve was drawn. (C) Tumor weights of the three groups were measured. Each dot represents one sample. (D) Comparison of body weight of C57 mice before and after C35 or PBS treatment. *p<0.05. **p<0.01.

As shown in Fig 6A–6C, despite the same number of Lewis cells injected, tumor growth in vivo was significantly reduced in either 50 mg/Kg C35 or 75 mg/Kg C35 treatment groups compared to PBS group, as evidenced by a decrease in tumor growth rate and weight of the excised tumor in C35 treated group compared to those in PBS group. Moreover, C35 did not cause a significant impairment in the bodyweight (Fig 6D) or tissue morphology of mice (S5 Fig in S1 File). All mice survived after treatment with C35 or PBS. In conclusion, consistent with the in vitro results, C35 had the antitumor activity in vivo.

## Discussion

Many natural and synthetic coumarin-like compounds have been extensively studied by many researchers for their anticancer activity due to their structural, non-toxic and biological properties [1]. These coumarin-based anticancer drugs have been identified through diverse mechanisms of action, such as alkylating agents [12, 13], topoisomerase inhibitors [14, 15], hormone antagonists [16–19], angiogenesis inhibitors [20–22], antimitotic agents [23–26], apoptosis inducers [27–31], human carbonic anhydrase inhibitors [32–35], telomerase inhibitors [36, 37]. Biscoumarin, a coumarin-derived compound, has been reported as a potent and efficient enzyme inhibitor as α-glucosidase inhibitor, α-amylase inhibitor, urease inhibitor, aromatase inhibitor [38]. Some studies have reported the development and biosynthesis of coumarin derivatives, and showed their anti-proliferative effects on tumor cells [3, 39–43], but the mechanism remains largely unknown.

Here, we synthesized biscoumarin C35 and found that C35 exhibited significant cytotoxicity against the lung cancer cells in a concentration dependent manner, but had little effect on normal cells. Moreover, C35 treatment in vivo did not cause parenchymal organ damage in brains, hearts, lungs, livers and kidneys. In addition, migration ability is related to the metastatic potential of cancer cells, which contributes to cancer progression and poor patient outcomes [44, 45]. Extracellular matrix and basement membrane are the main barriers for tumor metastasis [46]. Degradation of stromal collagen by MMP-2 and MMP-9 is the biochemical basis for tumor cell migration and invasion into surrounding tissues [47]. Therefore, the role of MMP-2 and MMP-9 is considered to be one of the key steps in tumor metastasis. Consistently, our results indicated that C35 dose-dependently inhibited the migration of H1299 and Lewis cells, which concomitant with the reduced expression of MMP-2 and MMP-9.

The MAPK signaling pathway has been reported to play an important role in cell proliferation, apoptosis and differentiation [48]. The p38 signaling pathway, one of the MAPK signaling pathways, plays a central role in regulating the expression and activity of MMPs [49, 50], and is integral in carcinogenesis and cancer maintenance [51, 52]. Activation of the p38 signaling pathway has been showed increases the expression of MMP-2 and MMP-9 [53]. In this study, we observed that C35 significantly repressed the phosphorylation of p38, indicating that C35 may exert its cytotoxic effect by inactivating p38 signaling pathway.

Furthermore, in our previous study [4] the synthetic biscoumarin 3,3’-((4-chlorophenyl)methylene)bis(4-hydroxy-2H-chromen-2-one) suppressed non-small cell lung cancer cell proliferation and induced cell apoptosis. Structure-activity relationship (SAR) research revealed that electron-withdrawing groups such as Cl and NO_2_ on benzaldehyde showed the most profound anti-cancer activity [54]. Mayank and colleagues [8] have developed furtherly that chloro- substituent at 2 or 6, or 4 position on benzaldehyde provided good anticancer activity. The rational design by the introduction of fluorine into a compound has achieved success in the development of organic anticancer drugs [5–7]. In our present study, although the similar biscoumarin C35 with 3,4,5-trifluoro substituents instead of 4-chloro on the benzaldehyde ring have higher IC50 value in Lewis lung cancer cells, biscoumarin C35 inhibited the migration of lung cancer cells. Most importantly, C35 showed anti-cancer capacity in vivo. It is worth for further study.

## Conclusions

In conclusion, our study showed that the biscoumarin C35 with 3,4,5-trifluoro substituents instead of 4-chloro on the benzaldehyde ring displayed strong cytostatic effects on lung cell proliferation, and also possessed a significant inhibition of migration by reducing the expression of MMP-2 and MMP-9 in lung cancer cells. Moreover, C35 treatment suppressed the phosphorylation of p38 in lung cancer cells, which may contribute to the anti-cancer activity of C35. Therefore, C35 may be a novel and effective approach for the treatment of lung cancer.

## Supporting information

S1 File (DOCX)

S2 File (XLSX)

S1 Raw images (PDF)
