# Supplementary material for: 3,3’-((3,4,5-trifluoropHenyl)methylene)bis(4-hydroxy-2H-chromen-2-one) inhibit lung cancer cell proliferation and migration
Source: PLoS One. 2024 May 22;19(5):e0303186. doi: 10.1371/journal.pone.0303186 (PMC11111047; doi:10.1371/journal.pone.0303186)
Supplement: S1 File — (DOCX) [file pone.0303186.s001.docx]

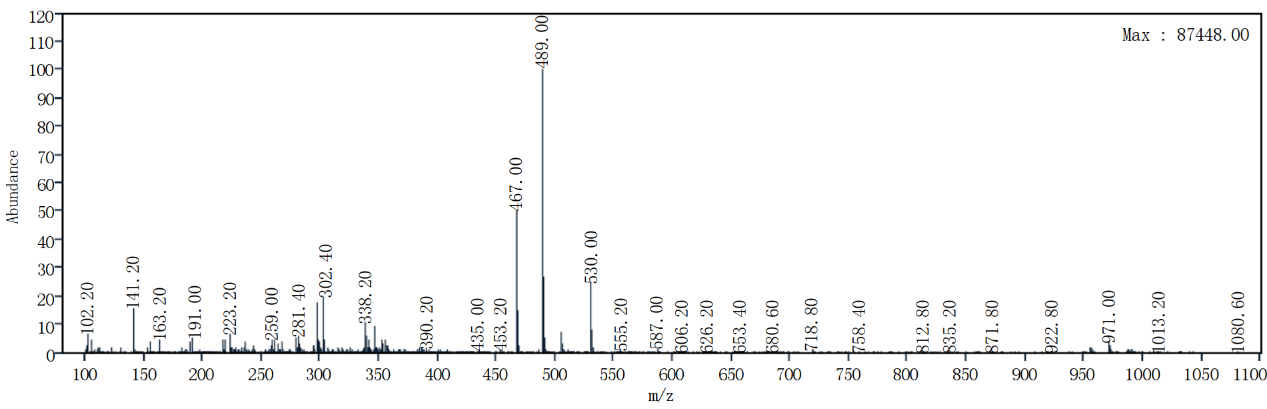


Fig S1. MS spectra of C35

Fig S2. IR spectra of C35


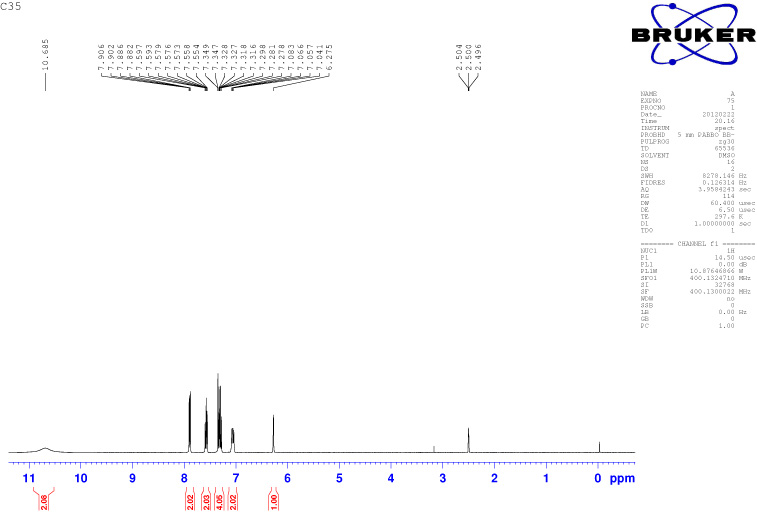


Fig S3. ^1^H NMR spectra of C35


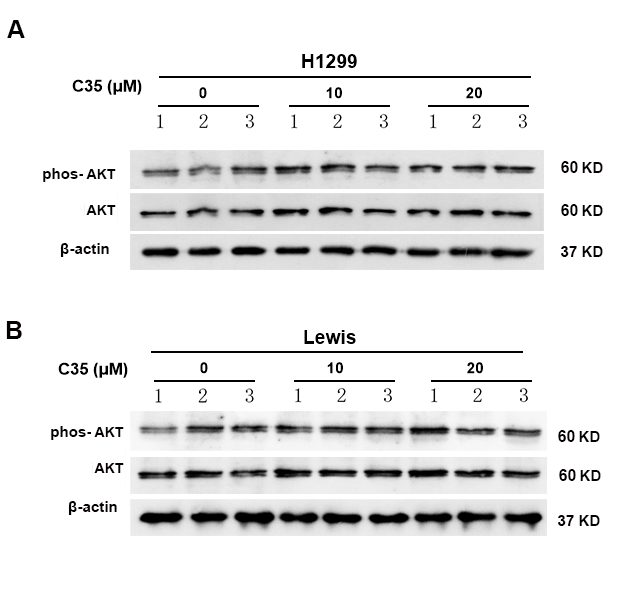


Fig S4. Western blot analyses of the phosphorylation of p38 in H1299 (A) and Lewis (B) cells treated with or without C35 as the indicated concentrations. β-actin served as loading control. The 1, 2 and 3 represent three of the samples in the indicated group.
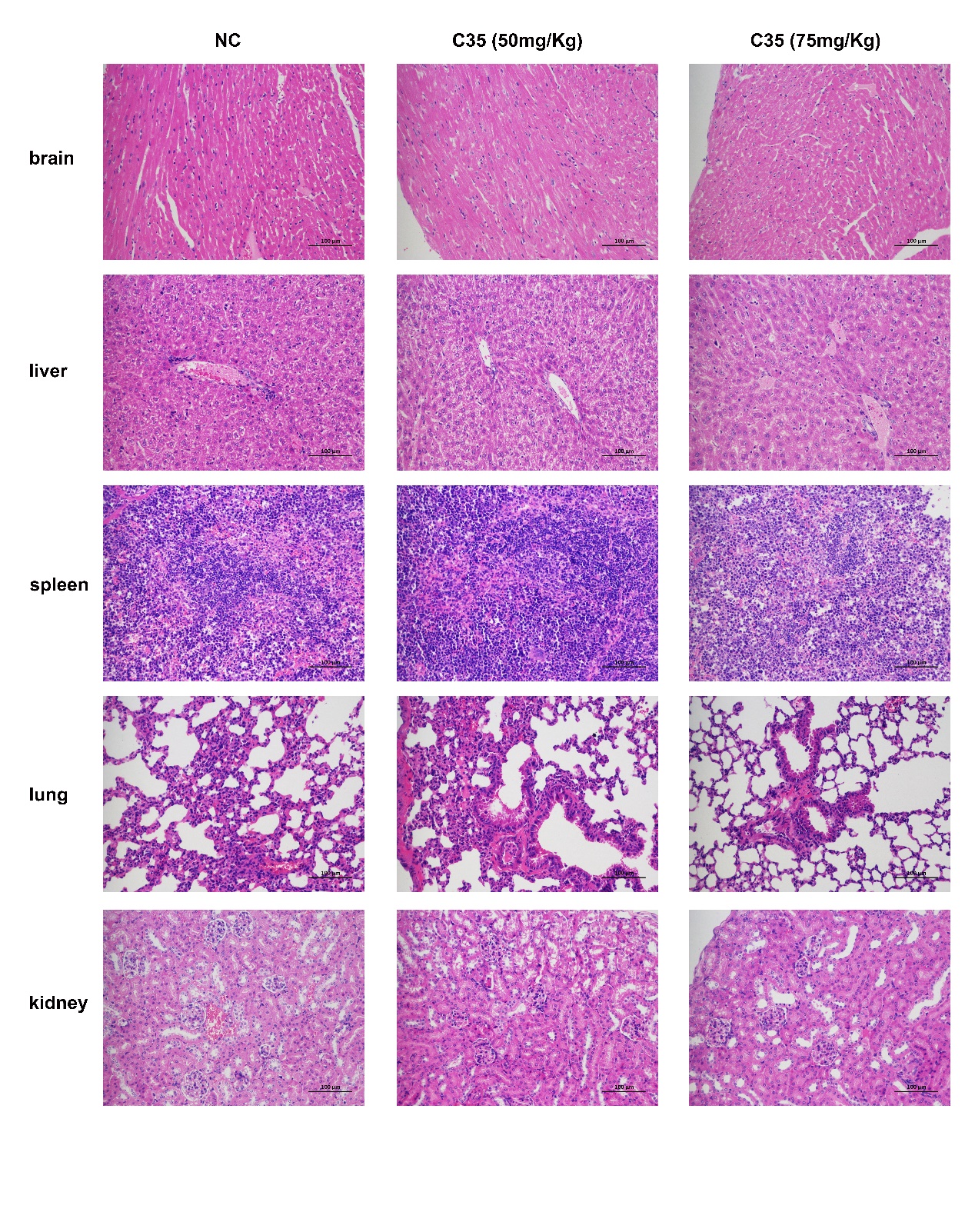


Fig S5. i.p. injection with C35 does not change the main tissue morphology.
