# Supplementary material for: 3,3’-((3,4,5-trifluoropHenyl)methylene)bis(4-hydroxy-2H-chromen-2-one) inhibit lung cancer cell proliferation and migration
Source: PLoS One. 2024 May 22;19(5):e0303186. doi: 10.1371/journal.pone.0303186 (PMC11111047; doi:10.1371/journal.pone.0303186)
Supplement: S1 Raw images — (PDF) [file pone.0303186.s003.pdf]

Fig 4A

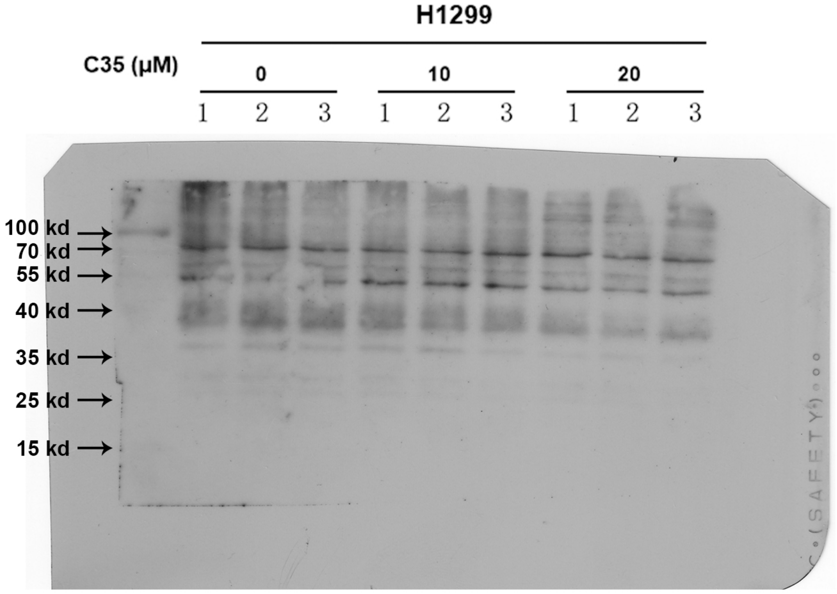

Antibody: MMP-2 SDS-PAGE 10% gel
Protein bands were detected on film

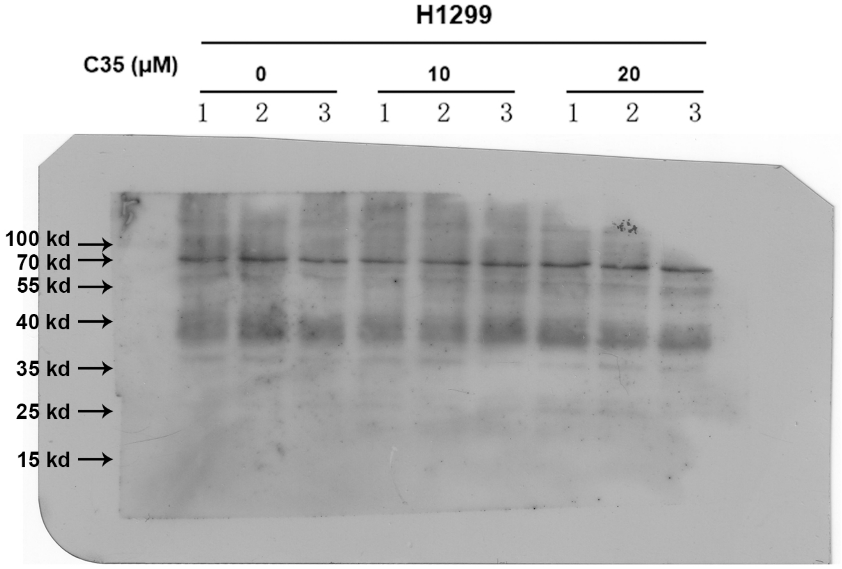

Antibody: MMP-9 SDS-PAGE 10% gel
Protein bands were detected on film

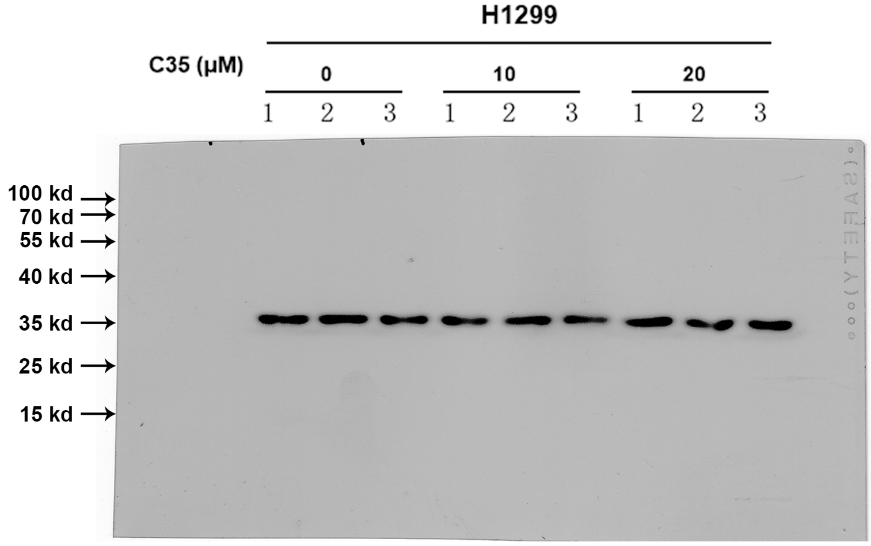

Antibody: β-actin SDS-PAGE 10% gel
Protein bands were detected on film

Fig 4B

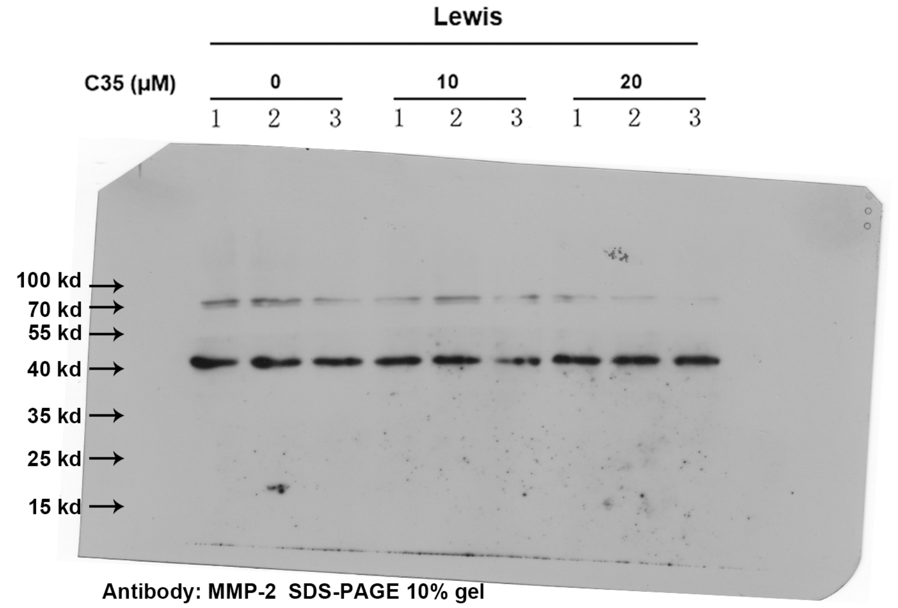

Antibody: MMP-2 SDS-PAGE 10% gel
Protein bands were detected on film

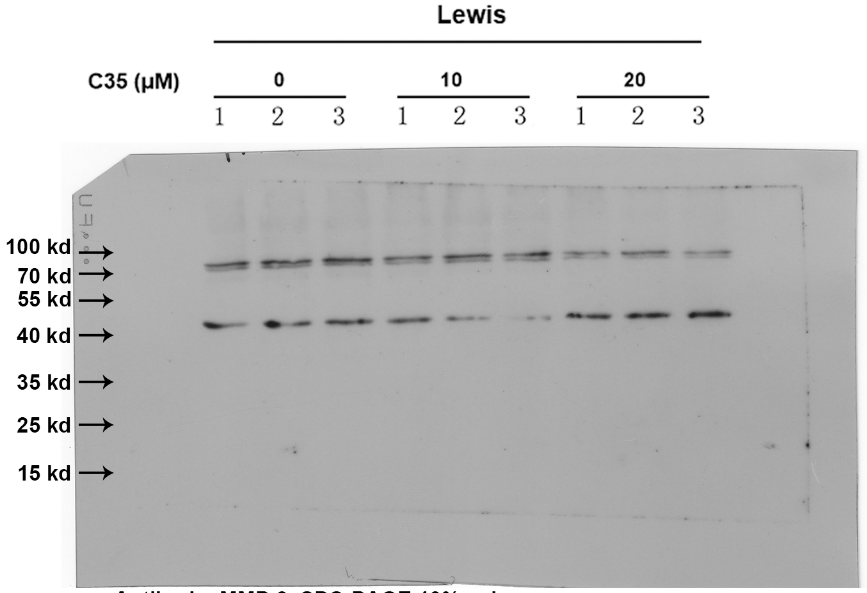

Antibody: MMP-9 SDS-PAGE 10% gel
Protein bands were detected on film

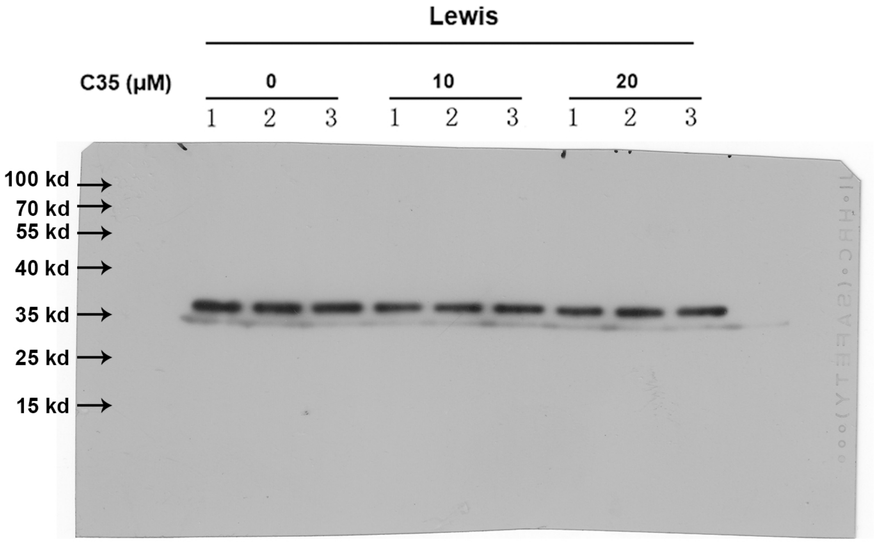

Antibody: β-actin SDS-PAGE 10% gel
Protein bands were detected on film

Fig 5A

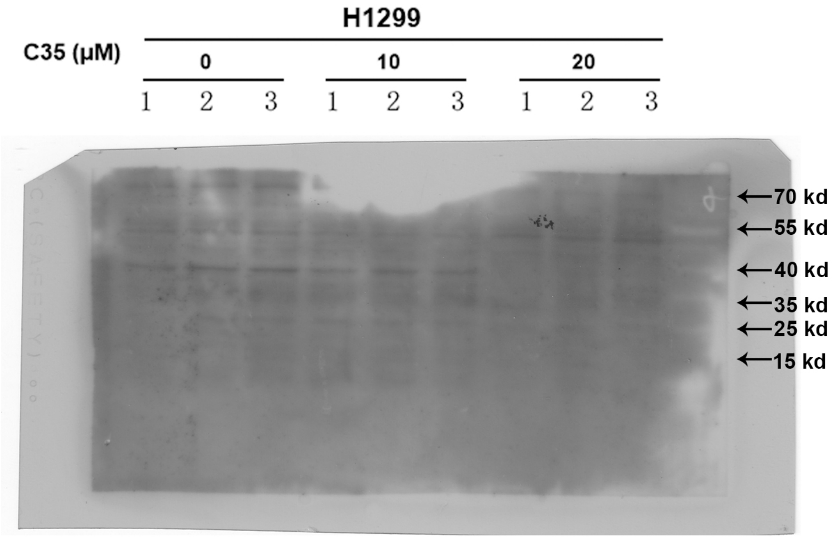

Antibody: phos-p38 SDS-PAGE 10% gel
Protein bands were detected on film

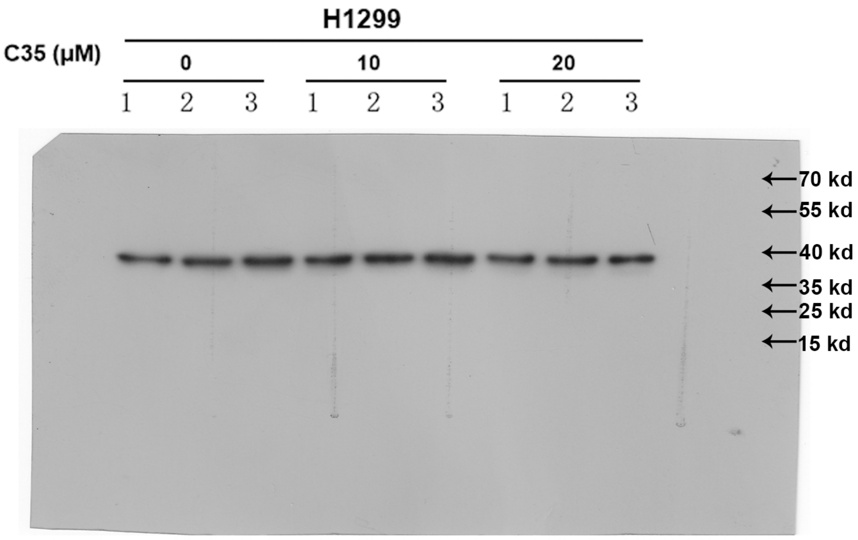

Antibody: p38 SDS-PAGE 10% gel
Protein bands were detected on film

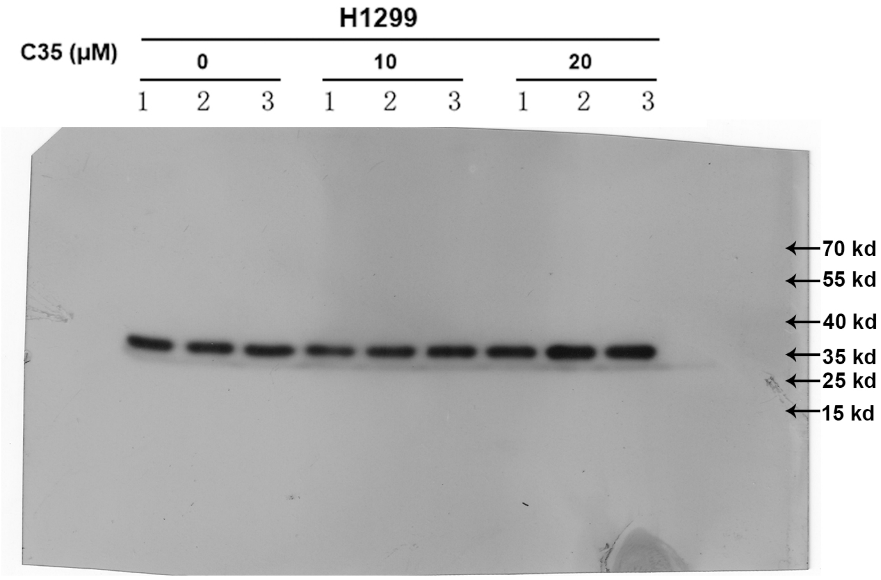

Antibody: β-actin SDS-PAGE 10% gel
Protein bands were detected on film

Fig 5B

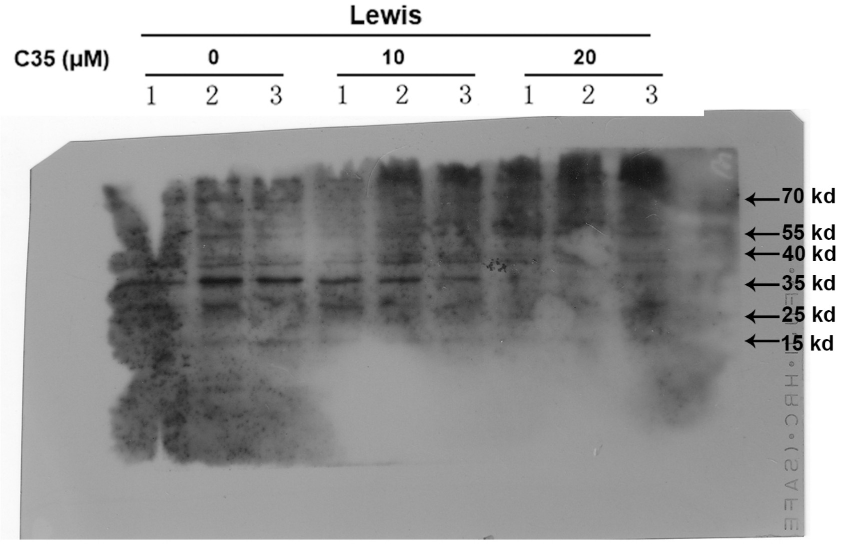

Antibody: phos-p38 SDS-PAGE 10% gel
Protein bands were detected on film

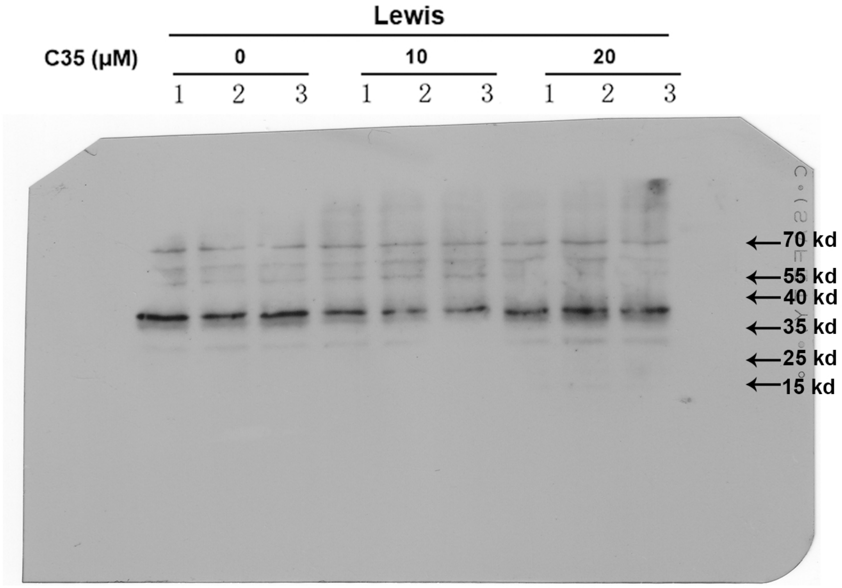

Antibody: p38 SDS-PAGE 10% gel
Protein bands were detected on film

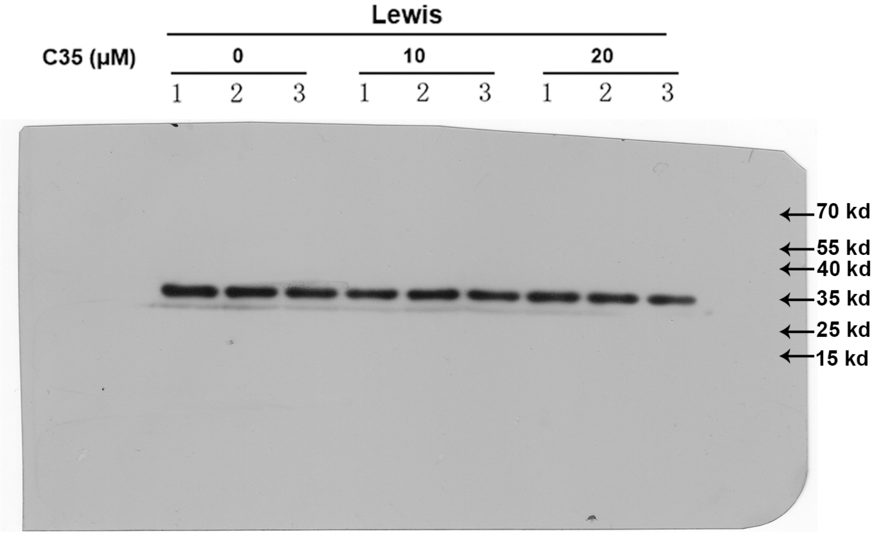

Antibody: β-actin SDS-PAGE 10% gel
Protein bands were detected on film

Fig S4A

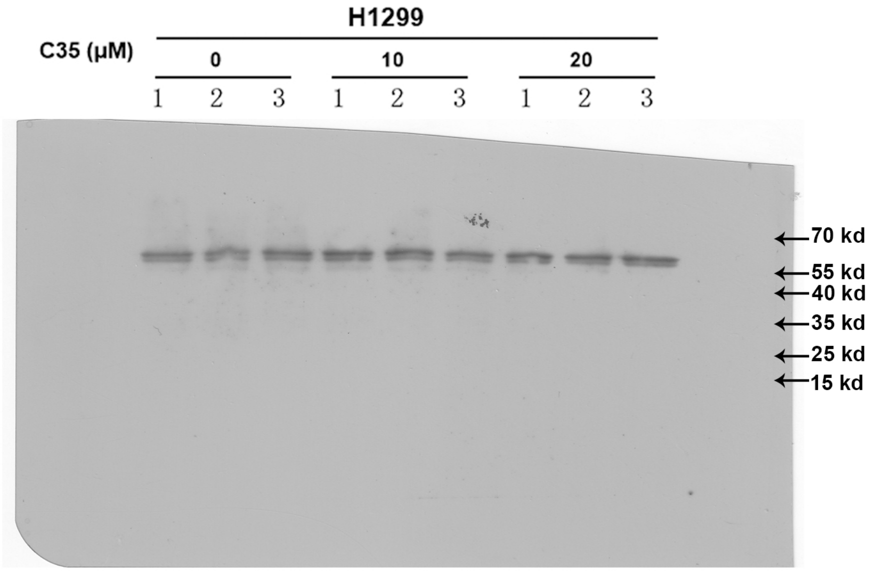

Antibody: phos-AKT SDS-PAGE 10% gel
Protein bands were detected on film

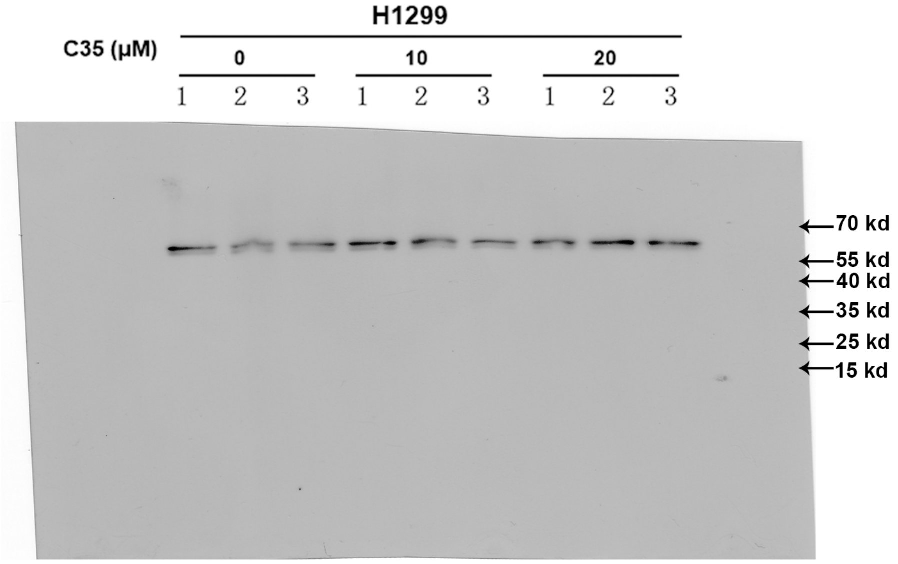

Antibody: AKT SDS-PAGE 10% gel
Protein bands were detected on film

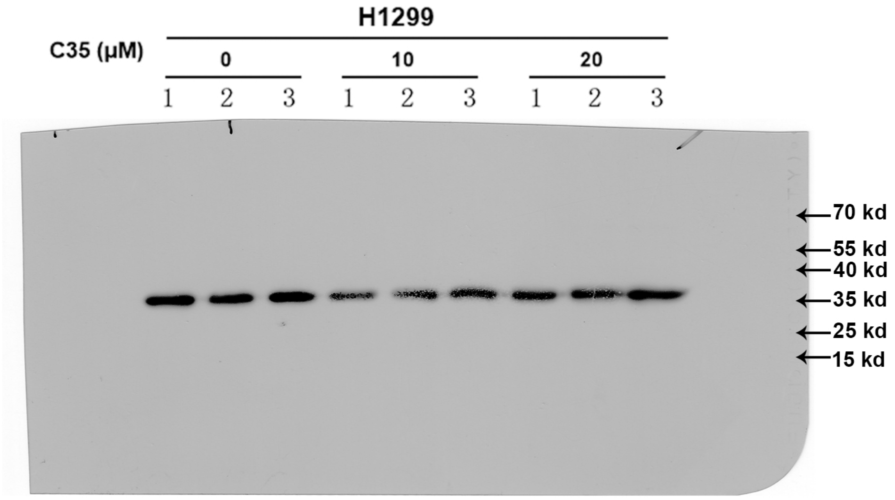

Antibody: β-actin SDS-PAGE 10% gel
Protein bands were detected on film

Fig S4B

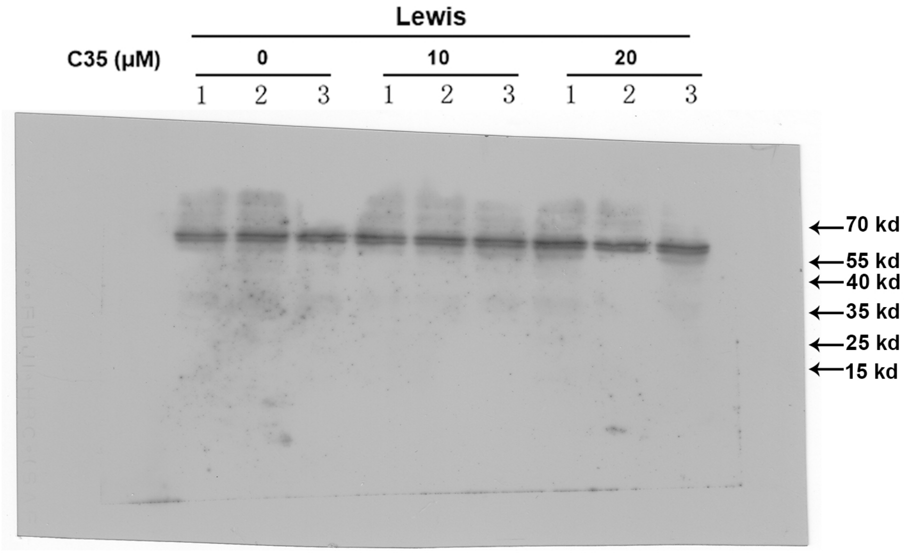

Antibody: phos-AKT SDS-PAGE 10% gel
Protein bands were detected on film

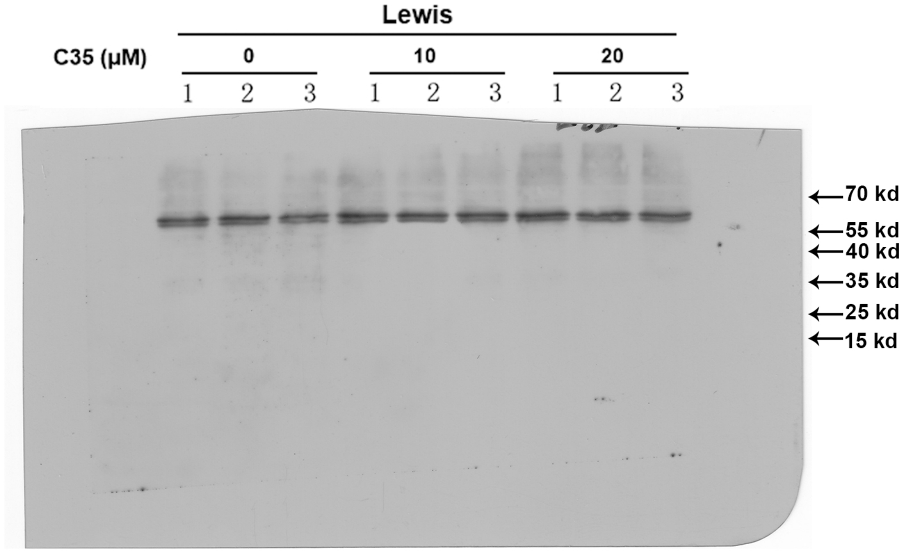

Antibody: AKT SDS-PAGE 10% gel
Protein bands were detected on film

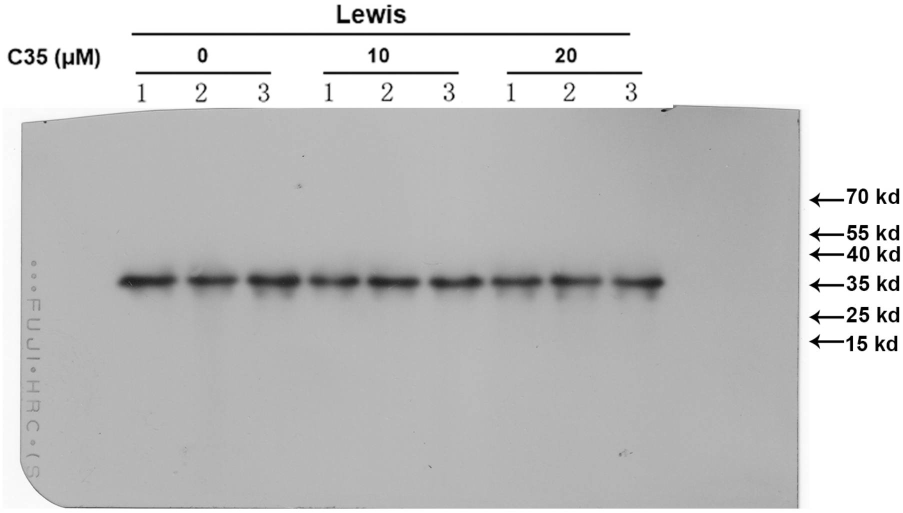

Antibody: β-actin SDS-PAGE 10% gel
Protein bands were detected on film
